# 人工智能识别技术在T1期肺癌诊断中的临床应用研究

**DOI:** 10.3779/j.issn.1009-3419.2019.05.09

**Published:** 2019-05-20

**Authors:** 晓鹏 刘, 海英 周, 志雄 胡, 权 金, 静 王, 波 叶

**Affiliations:** 1 201508 上海，复旦大学附属金山医院呼吸科 Department of Respiratory Disease, Jinshan Hospital of Fudan University, Shanghai 201508, China; 2 200030 上海，上海交通大学附属胸科医院胸外科 Department of Thoracic Surgery, Thoracic Hospital Affiliated to Shanghai Jiaotong University, Shanghai 200030, China

**Keywords:** 人工智能, 肺肿瘤, 计算机体层摄影, Artificial intelligence, Lung neoplasms, Computed tomography

## Abstract

**背景与目的:**

肺癌是目前国内外发病率及致死率最高的癌症，使用计算机断层扫描（computed tomography, CT）筛查肺癌结节工作量巨大。通过人工智能深度学习，在1 mm及5 mm层厚的胸部CT中，利用计算机人工智能自动寻找肺癌结节，以测试人工智能在肺癌自动识别中的效果。

**方法:**

分别标注5 mm及1 mm层厚的T1期肺癌患者胸部CT片各5, 000例，进行计算机神经网络学习，形成肺部结节的算法，利用人工智能形成的算法测试1 mm及5 mm层厚的T1期肺癌患者胸部CT片各500例，同人类读片进行比较，测试敏感性及特异性。

**结果:**

利用人工智能读取5 mm的胸部CT 500例，敏感度达95.20%，特异性达93.20%，两次重复读取的Kappa值达0.926, 1。对于1 mm的胸部CT 500例测试，敏感性为96.40%，特异性为95.60%，两次重复读取的Kappa值为0.938, 6。而与5位医师相比，对1 mm层厚的相同验证集CT片进行读片，人工智能与人工读片对于肺癌结节和阴性对照读片的检测率相似，两者之间比较无显著差异。而在5 mm层厚的相同验证集CT片比较中，人工智能对肺癌结节的检出数优于人工读片，敏感性更高，但误报数增多，特异性稍差。

**结论:**

通过人工智能自动学习早期肺癌胸部CT图像，可以达到较高的早期肺癌识别的敏感性及特异性，可辅助医生进行诊断工作。

## Abstract (en)

**Background and objective:**

Lung cancer is the cancer with the highest morbidity and mortality at home and abroad at present. Using computed tomography (CT) to screen lung cancer nodules is a huge workload. To test the effect of artificial intelligence in automatic identification of lung cancer by using artificial intelligence to find the lung cancer nodules automatically in the chest CT of 1 mm and 5 mm thick.

**Methods:**

5, 000 cases of T1 stage lung cancer patients with 1 mm and 5 mm layer thickness were respectively labeled and learned by computer neural network, the algorithm of forming pulmonary nodules was carried out. 500 cases of chest CT in T1 stage lung cancer patients with 1 mm and 5 mm thickness were tested by artificial intelligence formation, and the sensitivity and specificity were compared with artificial reading.

**Results:**

Using artificial intelligence to read chest CT 500 in 5 mm, the sensitivity was 95.20%, the specificity was 93.20%, and the Kappa value of two times repeated read was 0.926, 1. For 1 mm chest CT 500 cases, the sensitivity is 96.40%, the specificity is 95.60%, and the Kappa reads two times is 0.938, 6. Compared with 5 doctors, the same CT sets with 1 mm thickness were read. The detection rates of artificial intelligence and artificial reading were similar to those of lung cancer nodules and negative control read films, and there was no significant difference between them. In the comparison of the same CT slices with 5 mm thickness, the number of detection of lung cancer nodules by artificial intelligence is better than that of artificial reading, and the sensitivity is higher, but the number of false messages is increased and the specificity is slightly worse.

**Conclusion:**

The automatic learning of early lung cancer chest CT images by artificial intelligence can achieve high sensitivity and specificity of early lung cancer identification, and assist doctors in the diagnosis of lung cancer.

肺癌是目前国内外发病率及致死率最高的癌症，应用计算机断层扫描（computed tomography, CT）筛查发现早期肺癌病灶可降低肺癌的死亡率。随着CT扫描在各地人群中应用的增加，发现了许多可疑的实性结节（solid nodule）、部分实性结节（part-solid nodule）和磨玻璃密度结节（ground glass nodule, GGN），如何在大量的影像资料中区分出真正的早期肺癌病灶，成为影像及临床医生新的工作难点，将人工智能的方式应用到筛查肺部结节可能会有效地帮助解决这一难题。本研究将使用人工智能软件对10, 000例具有临床病理确诊的T1期肺癌患者CT片进行重复读取，评估人工智能在临床早期肺癌诊断中的准确性。

## 1 资料与方法

### 1.1 研究对象

选择2012年1月-2017年8月期间在上海交通大学附属胸科医院胸外科及复旦大学附属金山医院胸外科手术的T1期肺癌患者的CT片10, 000例作为人工智能学习集，其中1 mm和5 mm层厚CT片各5, 000例。

纳入标准：手术切除的T1期肺癌病灶，其诊断标准为：肺部CT影像提示1 cm≤结节直径≤3 cm，被肺或脏层胸膜包绕，未累及叶支气管近端以上位置，术后病理证实为肺恶性肿瘤^[15]^。排除标准：结节无手术切除病理学检查结果。剔除标准：术后病理提示为转移性肿瘤。

### 1.2 研究方法

#### 1.2.1 设备名称

definition AS，西门子128层螺旋CT。患者仰卧，头先进入，以胸骨柄为定位中心，患者深吸气并屏气；行胸部CT扫描，扫描参数调节：电流100 mA，电压120 kV，间距5 mm，层厚5 mm，矩阵512×512，螺距为0.993，范围为从肺尖以上扫描至横隔以下；发现病灶后给予靶向扫描，扫描参数：电流260 mA，电压120 kV，间距1 mm，层厚1 mm，矩阵不变，螺距调至1.375。

#### 1.2.2 人工标记

使用ITK-SNAP 3.4.0标记软件对T1期肺癌的胸部CT片进行人工标记。标记人员为具有读片经验10年以上的呼吸科主治及以上医师，结合术后病理报告、术前影像报告，对CT片上每个层面的肺癌结节病灶沿边缘精确圈标。

#### 1.2.3 肺癌结节的识别特征及分类规则学习

基于深度学习技术，应用前期与上海交通大学合作开发的人工智能系统^[16]^分别对1 mm和5 mm层厚CT图像中手工标出的肺癌样本集合进行训练，自动学习出肺癌结节的识别特征及分类规则。

#### 1.2.4 评价指标

根据95%目标特异性及敏感性，95%CI预估最小样本量为409例，故另外选择1 mm和5 mm层厚CT片各500例作为人工智能验证集，其中1 mm和5 mm层厚T1期肺癌胸部CT片各250例，1 mm和5 mm层厚正常影像CT片各250例，利用人工智能分别进行读片测试，重复2次读取结果评价一致性。同时选择上海交通大学附属胸科医院和复旦大学附属金山医院具有肺部读片经验10年以上的5位主治及以上医师对相同验证集CT片进行人工读片，每人使用随机双盲法抽读100例CT片，比较两种读片方法的特异性、敏感性，评价人工智能检测肺部小结节的有效性。

### 1.3 统计学处理

应用medcale统计软件，人工智能检测方法的真实性使用灵敏度、特异度来评价，并计算阳性似然比、阴性似然比，以阳性似然比≥10，阴性似然比≤0.10认为试验具有较高的诊断价值。使用Kappa分析评价人工智能检测方法的可靠性。以Kappa值=1表示完全一致，Kappa值< 0.4时一致性差，0.4-0.75为中、高度一致，Kappa值≥0.75为一致性极好。两种方法间率的比较采用卡方检验，以*P* < 0.05为有统计学差异。

## 2 结果

### 2.1 人工智能检测方法的真实性评价

人工智能筛查在1 mm层厚肺结节CT片中自动读片，自动寻找3 cm以下肺癌结节，敏感性为96.40%（95%CI: 93.28%-98.34%），特异性为95.60%（95%CI: 92.26%-97.78%），阳性似然比为21.91（95%CI: 12.29-39.06），阴性似然比为0.04（95%CI: 0.02-0.07），阳性似然比≥10，阴性似然比≤0.10，认为试验具有较高的诊断价值。见表 1。人工智能筛查在5 mm层厚肺癌结节CT片中自动读片，自动寻找3 cm以下肺癌结节，敏感性为95.20%（95%CI: 91.77%-97.50%），特异性为93.20%（95%CI: 89.34%-95.99%），阳性似然比为14（95%CI: 8.84-22.17），阴性似然比为0.05（95%CI: 0.03-0.09）。阳性似然比≥10，阴性似然比≤0.10，认为试验具有较高的诊断价值。见表 2。

**1:** 人工智能筛查肺癌结节（1 mm层厚） Artificial intelligence screening lung cancer nodules (1 mm)

Artificial intelligence screening	Lung nodules （*n*=250）	Nonodules （*n*=250）	Total
Positive (detection)	241	11	252
Negative (not detected)	9	239	248
Total	250	250	500

**2:** 人工智能筛查肺癌结节（5 mm层厚） Artificial intelligence screening lung cancer nodules (5 mm)

Artificial intelligence screening	Lung nodules （*n*=250）	Nonodules （*n*=250）	Total
Positive (detection)	238	17	255
Negative (not detected)	12	233	245
Total	250	250	

### 2.2 人工智能检测方法的可靠性评价

利用人工智能对1 mm层厚肺结节CT片进行读片测试，自动寻找3 cm以下肺癌结节，重复2次读取。结果显示，人工智能对1 mm层厚肺结节CT片2次读片Kappa值为0.938, 6，Kappa值接近1，提示一致性极好。见表 3。利用人工智能对5 mm层厚肺结节CT片进行读片测试，自动寻找3 cm以下肺癌结节，重复2次读取。结果显示，人工智能对5 mm层厚CT片2次读片Kappa值为0.926, 1，Kappa值接近1，提示一致性极好。见表 4。

**3:** 2次人工智能筛查肺癌结节kappa分析（1 mm层厚） Two times of artificial intelligence (AI) screening lung cancer nodules kappa analysis (1 mm)

The first AI screening	The second AI screening		Total
	Lung nodules（n=250）	Nonodules（n=250）	
Lung nodules（*n*=250）	238	9	247
Nonodules（*n*=250）	6	236	242
Total	244	245	489

**4:** 2次人工智能筛查肺癌结节kappa分析（5 mm层厚） Two times of artificial intelligence screening lung cancer nodules kappa analysis (5 mm)

The first AI screening	The second AI screening		Total
	Lung nodules（*n*=250）	No nodules（*n*=250）	
Lung nodules（*n*=250）	234	11	245
No nodules（*n*=250）	8	232	240
Total	242	243	485

### 2.3 将人工智能检测与人工读片进行比较

5位医师对1 mm层厚的相同验证集CT片进行人工读片，对CT片中3 cm以下肺癌结节诊断的敏感性为96.00%（95%CI: 92.77%-98.07%），特异性为98.40%（95%CI: 95.95%-99.56%），阳性似然比为60（95%CI: 22.69-158.67），阴性似然比为0.04（95%CI: 0.02-0.07）。见表 5。对5 mm层厚的相同验证集CT片进行人工读片，对CT片中3 cm以下肺癌结节诊断的敏感性为90.80%（95%CI: 86.52%-94.08%），特异性为97.20%（95%CI: 94.32%-98.87%），阳性似然比为32.43（95%CI: 15.61-67.39），阴性似然比为0.09（95%CI: 0.06-0.14），见表 6。人工智能与人工读片比较，检出率有显著差异，对于正常无结节阴性对照，检出准确率低于人工读片，而对于肺癌结节病灶，检出率高于人工读片（*P* < 0.05）。见表 7。在1 mm层面CT片上，人工智能读片与人工读片比较，检出率无显著差异（*P* > 0.05）。见表 7。

**5:** 人工读片筛查肺癌结节（1 mm层厚） Lung cancer nodules were screened by artificial reading (1 mm)

Artificial reading piece	Lung nodules （*n*=250）	No nodules （*n*=250）	Total
Positive (detection)	240	4	244
Negative (not detected)	10	246	256
Total	250	250	500

**6:** 人工读片筛查肺癌结节（5 mm层厚） Lung cancer nodules were screened by artificial reading (5 mm)

Artificial reading piece	Lung nodules （*n*=250）	No nodules （*n*=250）	Total
Positive (detection)	227	7	234
Negative (not detected)	23	243	266
Total	250	250	500

**7:** 人工智能筛查与人工读片比较 Comparison between artificial intelligence screening and artificial reading

	AI（1 mm）	Artificial reading piece（1 mm）		AI（5 mm）	Artificial reading piece (5 mm）	
Lung nodules（*n*=250）	241		240	238		227
No nodules（*n*=250）	239		246	233		243
Chi-square		0.139			14.807	
*P*		0.874^*^			0.006^#^	
Compared with manual film reading，^*^P < 0.05；Compared with manual film reading，^#^*P* < 0.05						

## 3 讨论

肺癌是目前国内外发病率及致死率最高的癌症，全世界每年约有60万的新发肺癌患者，低剂量CT筛查有助于早期发现肺癌，可降低20%的肺癌死亡率^[1, 2]^。胸部低剂量螺旋CT的概念于20世纪90年代初被首次提及，随之被应用于肺癌筛查中^[3, 4]^，目前已逐渐成为常规体检项目。

国内目前建议年龄在40岁以上或有长期吸烟史的肺癌高风险人群，每年至少接受一次胸部CT筛查，推广这种筛查工作的一个主要障碍是CT影像诊断的巨大工作量。早期肺癌多表现为肺部结节，它们尺寸小、对比度低、形状异质化高，目前绝大多数的医院仍是采用传统人工或结合CAD辅助读片的方式进行临床诊断，筛查工作主要由临床医生及影像医生人工完成。但是每位被检者的胸腔CT图像至少有100多张，精细级的扫描甚至多达600张，工作量巨大，且容易漏诊及误诊，所以随着体检人数的快速增长，人工处理的方法越来越难以胜任此项任务。

在过去的十多年里，多种针对肺部结节CT筛查的计算机辅助诊断（computer aided diagnosis, CAD）系统被开发出来，其中公开的有代表性的系统有ISICAD、SubsolidCAD、LargeCAD、ETROCAD等^[5-7, 8-14]^。这些CAD系统都是基于传统的机器视觉算法来检测肺部结节，目前主要应用于肺部大中结节的筛查，并且存在筛查特异性、敏感性均较低的情况^[6, 9, 14]^。近年来，市场上也开始研究人工智能应用于医疗行业的辅助诊断产品，但仍未形成行业技术标准，也未在临床进行推广。随着人工智能的发展，对于早期肺部小结节而言，通过人工智能产生的算法，辅助医生来阅片将是未来发展的方向。

本研究首先收集肺癌术后患者的CT，再结合术后病理报告和术前CT影像报告，精确找出肺癌病灶，再使用ITK-SNAP 3.4.0标记软件对肺癌结节沿边缘精确圈定，保证学习集的阳性金标准。我们的研究结果提示，利用人工智能读取5 mm的胸部CT 500例，对于3 cm以下肺癌结节的敏感度达到95.20%，特异性达93.20%，两次重复读取的Kappa值为0.926, 1，提示一致性极好。对于1 mm胸部CT 500例测试，敏感性为96.40%，特异性为95.60%，两次重复读取的Kappa值为0.938, 6，一致性极好。而与5位具有10年以上胸部CT阅片经验的高年资医师相比，同期对1 mm层厚的相同验证集CT片进行读片，人工智能与人工读片对于肺癌结节和阴性对照读片的检测率相似，两者之间比较无显著差异。而在5 mm层厚的相同验证集CT片比较中，人工智能对肺癌结节的检出数优于人工读片，敏感性更高，但误报数增多，特异性稍差，这是因为有经验的阅片人员，很少会对正常CT片产生误报，但由于5 mm层厚的CT片有可能无法展现早期肺癌的细节特征，故人工读片容易漏诊早期肺癌尤其是小于1 cm的病灶。我们将在未来通过不断增加样本量，通过人工智能自动学习特性，进一步改善人工智能筛查的特异性。另外，本项目的人工智能可达每秒30 mm的读片速度，平均10 s即可完成一份读片，大大节省了读片时间。因此，我们认为，通过人工智能自动学习早期肺癌胸部CT图像，可以达到较高的早期肺癌识别的敏感性及特异性，辅助医生进行诊断工作，减轻医生工作量。

将来随着训练样本的增多，我们研究的人工智能结节筛查率及准确性将不断得到提高。下一步我们将对结节进行亚分类检测，导入美国国立综合癌症网络（National Comprehensive Cancer Network, NCCN）指南，针对结节特性给出治疗建议，并且在此基础上，将人工智能模型嵌入型编程，拟将其制作成可以与PACS影像系统可插拔式对接的仪器，可直接服务于影像系统，即时获得影像资料并帮助医生及患者分析图像，并在临床验证该人工智能的有效性，在验证过程中不断完善其算法模型和与PACS系统对接的兼容性与多样性，并将其产业化，以期将此研究成果直接转化应用于临床。
